# LC-MS Analysis of the Polyphenolic Composition and Assessment of the Antioxidant, Anti-Inflammatory and Cardioprotective Activities of *Agastache mexicana* and *Agastache scrophulariifolia* Extracts

**DOI:** 10.3390/plants14142122

**Published:** 2025-07-09

**Authors:** Mihaela-Ancuța Nechita, Alina Elena Pârvu, Ana Uifălean, Sonia Iurian, Neli-Kinga Olah, Timea Henrietta Bab, Rodica Vârban, Vlad-Ionuț Nechita, Anca Toiu, Ovidiu Oniga, Daniela Benedec, Daniela Hanganu, Ilioara Oniga

**Affiliations:** 1 Department of Pharmacognosy, Faculty of Pharmacy, “Iuliu Hațieganu” University of Medicine and Pharmacy, Ion Creangă Street, 12, 400010 Cluj-Napoca, Romania; nechita_mihaela_ancuta@elearn.umfcluj.ro (M.-A.N.); dbenedec@umfcluj.ro (D.B.); dhanganu@umfcluj.ro (D.H.); ioniga@umfcluj.ro (I.O.); 2 Department of Morpho-Functional Sciences, Discipline of Pathophysiology, “Iuliu Haţieganu” University of Medicine and Pharmacy, 400012 Cluj-Napoca, Romania; parvualinaelena@umfcluj.ro (A.E.P.); uifaleanana@gmail.com (A.U.); 3 Department of Pharmaceutical Technology and Biopharmacy, Faculty of Pharmacy, “Iuliu Haţieganu” University of Medicine and Pharmacy, 41 V. Babes St., 400012 Cluj-Napoca, Romania; sonia.iurian@umfcluj.ro; 4 Department of Pharmaceutical Chemistry, Faculty of Pharmacy, “Vasile Goldiş” Western University of Arad, 310414 Arad, Romania; olah.neli@uvvg.ro; 5 PlantExtrakt Ltd., Rădaia, 407059 Cluj-Napoca, Romania; bab_timi@yahoo.com; 6 Department of Crop Science, Faculty of Agriculture, University of Agricultural Sciences and Veterinary Medicine of Cluj-Napoca, Calea Mănăștur Street 3–5, 400372 Cluj-Napoca, Romania; rodica.varban@usamvcluj.ro; 7 Department of Medical Informatics and Biostatistics, Faculty of Medicine, “Iuliu Hațieganu” University of Medicine and Pharmacy, Louis Pasteur Street 6, 400349 Cluj-Napoca, Romania; nechita.vlad@umfcluj.ro; 8 Department of Pharmaceutical Chemistry, Faculty of Pharmacy, “Iuliu Hațieganu” University of Medicine and Pharmacy, Victor Babeș Street 41, 400010 Cluj-Napoca, Romania; ooniga@umfcluj.ro

**Keywords:** *Agastache mexicana*, *Agastache scrophulariifolia*, polyphenols, tilianin, antioxidant, anti-inflammatory, cardioprotective

## Abstract

This study offers a detailed assessment of the polyphenolic composition and antioxidant, anti-inflammatory, and cardioprotective properties of lyophilized extracts derived from the aerial parts of *Agastache mexicana* and *Agastache scrophulariifolia*. The polyphenolic content was determined through the quantification of total polyphenols, flavonoids, and caffeic acid derivatives, complemented by LC-MS profiling. The antioxidant activity was evaluated in vitro using DPPH and FRAP assays, while the in vivo antioxidant and anti-inflammatory effects were investigated in a rat model of turpentine-oil-induced acute inflammation. Cardioprotective potential was assessed in a separate rat model of isoprenaline-induced myocardial infarction. Phytochemical analysis revealed a complex polyphenolic profile for both species, with tilianin and rosmarinic acid identified as predominant compounds. In the DPPH assay, both extracts exhibited marked radical scavenging activity (IC_50_: 65.91 ± 1.21 μg/mL for *A. mexicana*; 68.64 ± 2.48 μg/mL for *A. scrophulariifolia*). In the in vivo assays, the administration of the extracts significantly decreased pro-oxidant biomarkers (TOS, OSI, MDA, NO) and enhanced antioxidant markers (TAC, SH groups). Furthermore, the extracts led to a significant reduction in serum levels of GOT, GPT, and CK-MB in rats subjected to myocardial injury, supporting their cardioprotective efficacy. Overall, the results suggest that *A. mexicana* and *A. scrophulariifolia* represent promising natural sources of polyphenolic compounds with potential therapeutic value in oxidative-stress-related inflammatory and cardiovascular disorders.

## 1. Introduction

Inflammation represents a complex biological response of the immune system to a variety of stimuli, both infectious and non-infectious, including cellular damage, toxic agents, and irradiation. The activation of inflammatory pathways initiates a cascade of chemical signals that mediate leukocyte recruitment from the systemic circulation to the site of injury. These immune cells release cytokines and other molecular mediators that orchestrate the inflammatory response [1]. Chronic, low-grade inflammation has been increasingly recognized as a critical contributor to the pathogenesis of cardiovascular disease (CVD) [2]. Recent studies suggest that inflammatory markers may provide a more accurate assessment of cardiovascular risk than traditional lipid profiles, such as elevated low-density lipoprotein (LDL) cholesterol [3]. For example, elevated levels of C-reactive protein (CRP), a well-established marker of systemic inflammation, have been correlated with increased incidence of myocardial infarction and stroke [4,5,6,7]. Furthermore, chronic inflammatory disorders—such as rheumatoid arthritis, psoriasis, and systemic lupus erythematosus—have been linked to higher cardiovascular risk, attributable to persistent systemic inflammation in conjunction with conventional risk factors [8].

Ischemic heart disease, most commonly resulting from coronary artery atherosclerosis or functional abnormalities in coronary circulation, can be managed through lifestyle modifications, pharmacological intervention, and revascularization procedures [9]. However, emerging evidence indicates that myocardial infarction may result from diverse etiologies beyond atherosclerotic plaque rupture. Various pathophysiological mechanisms—such as metabolic dysregulation, inflammation, oxidative stress due to the overproduction of reactive oxygen species (ROS), and impaired antioxidant defense systems—may contribute to myocardial injury. These findings underscore the necessity of exploring therapeutic strategies targeting both oxidative stress and inflammatory processes [10].

In this context, phytochemicals have garnered considerable interest due to their antioxidant and anti-inflammatory properties, which may contribute to cardiovascular protection [11,12]. A number of plant-derived bioactive compounds with cardioprotective effects have been identified, including diosgenin, sulforaphane, catechin, quercetin, and tilianin [13,14]. Among these, tilianin—a flavonoid glycoside—has demonstrated cardioprotective activity in both in vitro and in vivo models. Its mechanisms of action include reduction in mitochondrial calcium and ROS levels, malondialdehyde (MDA), lactate dehydrogenase (LDH), and creatine kinase-MB (CK-MB) levels, while enhancing the levels of ATP, NAD, and superoxide dismutase (SOD) [15,16]. Tilianin is present in several medicinal plants, including species of the *Agastache* genus, where it constitutes a major component of the extracts [14,17,18,19,20].

The *Agastache* genus (Clayton ex Gronov), belonging to the Lamiaceae family, comprises approximately 22 aromatic species distributed across North America and East Asia. These plants are traditionally valued for both medicinal and ornamental purposes [21,22]. *Agastache mexicana* (Kunth) Lint & Epling (AM), commonly referred to as “toronjil” or lemon balm, is native to Mexico and is traditionally used for its anxiolytic and sedative properties, as well as in empirical treatments for heart conditions [23]. *Agastache scrophulariifolia* (Willd.) Kuntze (AS), also known as purple giant hyssop, is distributed throughout North America and parts of Canada. Despite its traditional usage, the phytochemical composition and biological activities of *A. scrophulariifolia* remain insufficiently characterized, highlighting the need for further pharmacognostic and pharmacological investigations.

Given the growing interest in the therapeutic potential of medicinal plants and the ethnopharmacological relevance of *Agastache* species, the present study aimed to evaluate the polyphenolic profile and assess the antioxidant, anti-inflammatory, and cardioprotective activities of lyophilized extracts obtained from the aerial parts of *A. mexicana* and *A. scrophulariifolia*. To the best of our knowledge, this study provides one of the first detailed investigations into the polyphenolic profile of *A. scrophulariifolia* extract, alongside an in vivo evaluation of its anti-inflammatory and cardioprotective effects, thereby contributing to the limited body of existing research on this species.

## 2. Materials and Methods

### 2.1. Plant Material and Extraction Procedure

The aerial parts of *A. mexicana* and *A. scrophulariifolia* were collected during the flowering stage (principal growth stage 6, secondary growth stage 65) from the experimental field of the University of Agricultural Sciences and Veterinary Medicine, Cluj-Napoca (46°45′33.1″ N, 23°34′27.7″ E) [24]. Voucher specimens were deposited in the Department of Pharmacognosy, Faculty of Pharmacy, Cluj-Napoca, Romania, under reference numbers 134 (AS) and 141 (AM). The plant material was air-dried at ambient temperature, ground to a fine powder using an electric grinder, and subsequently used for extraction.

Extraction was performed using powdered plant material and 50% (*v*/*v*) ethanol in water, with a plant-to-solvent ratio of 1:10 (*m*/*v*), at 60 °C for 30 min in a water bath. After filtration using filter paper, a part of each extract was used for phytochemical determinations, and the other part was concentrated under reduced pressure using a rotary evaporator (Hahnvapor, HS-2000NS, manufactured by Hahnshin Scientific Co., Ltd., Bucheon-si, Gyeonggi-do, South Korea), and was subjected to a lyophilization (freeze-drying) process.

### 2.2. Freeze-Drying of Extracts

The concentrated extracts were freeze-dried using a VirTis Advantage Plus Freeze Dryer (SP Scientific, Gardiner, MT, USA). The samples were initially frozen at −55 °C for 12 h, followed by primary drying conducted at −25 °C under a pressure of 0.2 mbar for 24 h. Subsequently, secondary drying was performed at 20 °C for an additional 12 h. The yield of lyophilization was 19.48% for AS extract and 21.56% for AM extract. The resulting lyophilized products were stored in a desiccator to prevent moisture absorption and preserve their physicochemical stability prior to in vivo pharmacological determinations.

### 2.3. Determination of Total Polyphenolic, Flavonoid and Caffeic Acid Derivatives Content

The total polyphenolic content (TPC), total flavonoid content (TFC), and total caffeic acid derivatives content (TCADC) in the two ethanolic extracts were determined using validated spectrophotometric methods adapted from both the Romanian and European Pharmacopoeias. Specific colorimetric reagents—Folin–Ciocalteu for TPC, aluminum chloride (AlCl_3_) for TFC, and Arnow’s reagent for TCADC—were employed.

For the quantification of TPC, 2 mL of diluted sample was mixed with 1 mL of Folin–Ciocalteu reagent, 10 mL distilled water, and 29% sodium carbonate to reach a final volume of 25 mL. After 30 min of incubation in the dark, absorbance was measured at 760 nm. TPC was calculated based on a calibration curve of gallic acid (concentrations ranging from 10 to 200 µg/mL; R^2^ = 0.999) and expressed as mg GAE per g dried plant material.

To determine the TFC, 5 mL of extract was combined with 5 mL of 10% sodium acetate and 3 mL of 25% AlCl_3_, then adjusted to 25 mL with methanol in a volumetric flask. The absorbance was recorded at 430 nm, and TFC was derived from a rutin standard curve (concentrations ranging from 10 to 80 µg/mL; R^2^ = 0.992), results being expressed as mg RE per g of dried plant material.

TCADC was assessed by reacting 1 mL of each extract with 1 mL of 0.5 N HCl, 1 mL of freshly prepared Arnow’s reagent (containing 10 g sodium nitrite and 10 g sodium molybdate in 100 mL water), and 1 mL of sodium hydroxide. The mixture was brought to 10 mL with distilled water in a calibrated flask. After homogenization, absorbance was read at 500 nm. TCADC was calculated using a calibration curve generated with caffeic acid (concentrations ranging from 5 to 100 µg/mL; R^2^ = 0.989) and expressed as mg of CAE per g of dried plant material. All determinations were performed in triplicate to ensure reproducibility and accuracy [25].

### 2.4. LC-MS Analysis

The Shimadzu Nexera I LC/MS-8045 UHPLC system (Kyoto, Japan) was used for the analysis. The system included a quaternary pump, autosampler, ESI probe, and triple quadrupole mass spectrometer.

Chromatographic separation was performed using a Phenomenex Luna C18 reversed-phase column (150 mm × 4.6 mm × 3 mm, 100 Å) (Torrance, CA, USA). The column temperature was maintained at 40 °C throughout the analysis. The mobile phase consisted of a gradient of methanol (Merck, Darmstadt, Germany) and ultrapure water produced using the Simplicity Ultra-Pure Water Purification System (Merck Millipore, Billerica, MA, USA). The gradient composition is detailed in Table 1. Formic acid (Merck, Darmstadt, Germany) was used as organic acid pH modifier. Both methanol and formic acid were of LC-MS grade. The flow rate was set to 0.5 mL/min.

A targeted LC-MS/MS method was employed for the quantitative determination of polyphenolic compounds. The analysis was performed using a triple quadrupole mass spectrometer operated in electrospray ionization (ESI) negative mode with multiple reaction monitoring (MRM). The interface temperature was maintained at 300 °C, and the capillary voltage was set to +3000 V. Nitrogen was used for both vaporization and as drying gas, at 35 psi and 10 L/min, respectively. An injection volume of 1 μL was used for each reference standard and was kept constant across all concentrations. MRM analysis employed compound-specific transitions, with quantifier and qualifier ions selected based on optimization using analytical standards. Retention times for each compound were confirmed with reference standards and served as an additional parameter for identification. Calibration curves were constructed using these standards for the quantification of the compounds. Each sample was injected in triplicate to ensure reproducibility [26].

### 2.5. Evaluation of In Vitro Antioxidant Activity

#### 2.5.1. DPPH Radical Scavenging Activity

The DPPH assay was used to evaluate the radical scavenging capacity of the extracts. A volume of 2 mL of each dilution (prepared with extract volumes ranging from 0.25 mL to 2.00 mL, adjusted to 2 mL with methanol) was mixed with 2 mL of 0.1 g/L DPPH methanolic solution. After incubation for 30 min at 40 °C in a thermostatic bath, absorbance was measured at 517 nm. Antioxidant activity (AA%) was calculated using the following formula:

AA% = [(A_control − A_sample)/A_control] × 100

where *A_control* represents the absorbance of the DPPH solution without extract, and *A_sample* the absorbance with extract. Results were expressed using the half-maximal inhibitory concentration (IC_50_) (µg/mL), and all determinations were performed in triplicate [25].

#### 2.5.2. Ferric-Reducing Antioxidant Power (FRAP) Assay

A FRAP assay was conducted to measure the antioxidant potential of the extracts based on their ability to reduce Fe^3+^ to Fe^2+^ in the presence of 2,4,6-tripyridyl-s-triazine (TPTZ). The FRAP reagent was freshly prepared by mixing 25 mL of acetate buffer (pH 3.6), 2.5 mL of 10 mM TPTZ in 40 mM HCl, and 2.5 mL of 20 mM ferric chloride solution. Each sample (0.4 mL) was diluted to 1.8 mL with distilled water and added to 6 mL of FRAP reagent. A blank was prepared using water instead of sample. Absorbance was measured at 450 nm. Antioxidant activity was expressed as µM of Trolox equivalents (TEs) per 100 mL of extract. All measurements were conducted in triplicate [25].

### 2.6. In Vivo Studies

#### 2.6.1. Experimental Animals

A total of 90 adult male Wistar albino rats (200–250 g) were used in the study, with 50 allocated to the first experimental series and 45 to the second. A single control group (n = 5) was shared between both experiments to reduce the number of animals used, in accordance with ethical principles. The choice of a minimum of 5 animals per group was made based on ethical considerations, in accordance with the principles of reduction and animal welfare, without compromising the statistical relevance of the results. Animals were bred and maintained at the Animal Facility of the Iuliu Hațieganu University of Medicine and Pharmacy, Cluj-Napoca, under standard laboratory conditions (12 h light/dark cycle, 21–22 °C temperature, 50–55% relative humidity), with ad libitum access to a pellet-based standard diet (Cantacuzino Institute, Bucharest, Romania). At the end of the experiments, animals were euthanized by cervical dislocation under general anesthesia with ketamine (60 mg/kg b.w.) and xylazine (15 mg/kg b.w.). All animals were monitored daily for general behavior, signs of lethargy, pain, or distress. All procedures involving animals were conducted in accordance with the European Union Directive 2010/63/EU on the protection of animals used for scientific purposes and the experimental protocol was approved by the Ethics Committee of Iuliu Hațieganu University of Medicine and Pharmacy, Cluj-Napoca (No. AVZ260/04.10.2023), as well as by the National Sanitary Veterinary and Food Safety Authority (No. 389/23.11.2023). A research protocol including the study design, objectives, and procedures was submitted and approved by the university’s Ethics Committee prior to the start of the experiment.

Animals were distributed into experimental groups by simple random allocation, without applying a formal randomization method or sequence generation. To minimize potential confounding factors, treatments were administered daily at the same hour and in a consistent group order throughout the experiment. The researcher conducting the experiment, outcome assessment, and data analysis was aware of the group allocation at all stages of the study.

#### 2.6.2. Investigation of In Vivo Anti-Inflammatory Activity

Prior to the beginning of the experiment, treatment solutions were prepared by dissolving specific amounts of lyophilized extract in distilled water, as follows: AS1 (19 mg lyophilized extract/mL water), AS2 (9.5 mg/mL), AS3 (4.75 mg/mL), AM1 (21 mg/mL), AM2 (10.5 mg/mL), AM3 (5.25 mg/mL).

A total of 50 animals (n = 5/group) were randomly assigned to 10 groups. Except for the negative control group (CONTROL), all groups received an intramuscular injection of turpentine oil (6 mL/kg body weight) on day 1 to induce inflammation. Simultaneously, oral treatments were administered daily by gavage for 10 consecutive days as follows: CONTROL and inflammation (INFL) groups received distilled water (1 mL/rat/day); AM groups received lyophilized extract in three concentrations (AM1, AM2, AM3) at 1 mL/rat/day; AS groups received the same dosing regimen (AS1, AS2, AS3); the reference drug group received diclofenac (10 mg/kg body weight/day, DICLO); and the antioxidant control group received Trolox (50 mg/kg body weight/day). On day 11, animals were anesthetized with ketamine (60 mg/kg b.w.) and xylazine (15 mg/kg b.w), and blood samples were collected via retro-orbital plexus. Serum was separated and stored at −80 °C for subsequent analysis [27,28]. All animals initially included in the experiment (n = 5 per group) were monitored throughout the study period. Data from all animals were included in the statistical analysis, and no exclusions were necessary.

#### 2.6.3. Evaluation of In Vivo Cardioprotective Activity

This experiment initially included 5 animals per group, for a total of 45 animals across 9 experimental groups: (1) negative control (CONTROL); (2) isoprenaline (ISO); (3) ISO + Trolox (50 mg/kg/day); (4–6) AM extract groups (AM1, AM2, AM3); and (7–9) AS extract groups (AS1, AS2, AS3). All treatments were administered orally (1 mL/rat/day) for 7 days. Myocardial infarction (MI) was induced on days 8 and 9 by subcutaneous administration of isoprenaline (150 mg/kg body weight), except in the CONTROL group. On day 10, under general anesthesia (ketamine 60 mg/kg b.w. + xylazine 15 mg/kg b.w.), blood samples were collected via retro-orbital puncture. Serum was separated and stored at −80 °C for subsequent analysis of cardiac biomarkers (CK-MB, GOT, GPT) using commercial diagnostic kits [28,29]. A total of 16 animals (Group ISO: 1, ISO + Tolox: 2, AS2: 3, AS3: 3, AM1: 3, AM2: 1, AM3: 3) did not complete the study due to mortality and were therefore excluded from the final statistical analysis.

#### 2.6.4. Measurement of Oxidative Stress Parameters

Oxidative stress parameters were measured spectrophotometrically using a Jasco V-350 UV-VIS spectrophotometer (Jasco International Co., Tokyo, Japan). Total oxidative status (TOS) was evaluated by a colorimetric method based on the oxidation of ferrous to ferric ions in the presence of oxidants, and expressed as H_2_O_2_ equivalents (µmol/L). Total antioxidant capacity (TAC) was assessed using the method of Erel and expressed as mmol of Trolox equivalents/L. The oxidative stress index (OSI) was calculated as the ratio of TOS to TAC. Malondialdehyde (MDA) levels were determined using the thiobarbituric acid reactive substances (TBARS) assay and reported as µM/L. Nitric oxide (NO) levels were quantified using the Griess reagent and expressed as µM/L. Total thiol content (SH) was measured using Ellman’s reagent and expressed as µM/L [28,29].

### 2.7. Statistical Analysis

The analysis was conducted using RCMDR version 4.5.0 (The R Project for Statistical Computing), with data collected in a Microsoft Excel 2016 database. Results are presented as mean ± standard deviation (SD). Comparisons were performed based on data distribution: Student’s *t*-test was used for normally distributed data, and the Mann–Whitney *U* test was used for non-normally distributed data. The Shapiro–Wilk test, appropriate for small sample sizes, was employed to assess normality. A *p*-value of <0.05 was considered statistically significant.

## 3. Results and Discussion

### 3.1. Phytochemical Analysis by LC-MS

The phytochemical composition of the two ethanolic extracts was determined by LC-MS [30] and is presented in Table 2, with compound concentrations expressed as μg/mL of extract.

The LC-MS profiles of both AM and AS extracts revealed a similar composition, including a total of 24 polyphenolic identified compounds. These comprised five glycosylated flavonoids (tilianin, luteolin-7-O-glucoside, vitexin, hyperoside, rutoside), nine flavonoid aglycones (acacetin, apigenin, kaempferol, chrysin, luteolin, myricetin, naringenin, hesperetin, quercetin), seven phenolic acids (rosmarinic acid, chlorogenic acid, caffeic acid, trans-*p*-coumaric acid, ellagic acid, gallic acid, salicylic acid), two diterpenes (carnosol and carnosic acid), and one coumarin derivative (esculetin).

Tilianin was identified as the major compound in both extracts, followed by rosmarinic acid and chlorogenic acid. Notably, AS extract had a higher tilianin concentration (55,574.8 ± 411.21 μg/mL) compared to AM (51,635.8 ± 278.21 μg/mL). Conversely, the AM extract contained greater amounts of rosmarinic acid (5910.3 ± 99.41 μg/mL) and chlorogenic acid (4005.2 ± 35.42 μg/mL) than AS (4263.3 ± 59.24 μg/mL and 3362.3 ± 5.47 μg/mL, respectively). Other compounds found in relatively high concentrations in both extracts included kaempferol, luteolin-7-O-glucoside, and esculetin.

Our results are in accordance with those published by other authors, who showed that AM extract contained tilianin, acacetin, and (2-acetyl)-7-O-glucosyl acacetin as the major compounds [31]. In addition, tilianin has been reported as the main component in other *Agastache* sp. extracts, including *A. rugosa*. Hwang et al. reported a content of 21.14 mg/g tilianin and 9.94 mg/g acacetin [32], while others obtained values of 38.29 mg/g tilianin and 7.88 mg/g acacetin for *A. rugosa* extract [33]. Another study conducted by our team quantified the level of tilianin in *A. rugosa* and *A. foeniculum* extracts. We found 5038.48 μg/mL of tilianin in *A. rugosa* and 2394.02 μg/mL of tilianin in *A. foeniculum* [34], suggesting that compound levels may vary depending on species and harvest season.

Regarding the levels of rosmarinic acid and chlorogenic acid, other authors have demonstrated that rosmarinic acid was one of the primary constituents of *A. rugosa* ethanolic [17], aqueous [35], and methanolic extracts [36], detected at a concentration of 25.81 mg/g in the ethanolic extract [33]. Another study found that the concentration of rosmarinic acid in *A. rugosa* varied with plant organ and developmental stage, with flowers having the highest concentration (48.43 μg/g), followed by roots (30.97 μg/g) and leaves (22.14 μg/g) [37]. In addition, our previous study proved that *A. rugosa* extract contained 2396.2 μg/mL chlorogenic acid and 4608.3 μg/mL rosmarinic acid, while *A. foeniculum* extract had 1859.9 ± 21.71 μg/mL chlorogenic acid and 3763.3 ± 57.84 μg/mL rosmarinic acid [34].

To date, no specific data have been reported in the available literature concerning the tilianin and rosmarinic acid content in *A. scrophulariifolia*. Therefore, the originality of our study lies in the identification and quantification of the major components of the extract, with our findings demonstrating the presence of twenty-four distinct polyphenolic compounds in the ethanolic extract of *A. scrophulariifolia*.

Tilianin, the primary component of both extracts, is a glucoside derivative of acacetin that has been found to exhibit cardioprotective effects in vitro and in vivo. According to current research, tilianin is a promising natural lead molecule for cardiovascular diseases such as ischemic heart disease and hypertension [14]. Tilianin’s protective properties are linked to a range of activities, which include free radical scavenging, anti-inflammatory actions, mitochondrial function regulation, and modulation of several signaling pathways [14,15,16,38].

Rosmarinic acid exhibits significant anti-inflammatory and antioxidant properties, exerting its effects by downregulating the expression of pro-inflammatory cytokines and mediators [39]. Studies have demonstrated that its anti-inflammatory activity is partly mediated through the inhibition of key signaling pathways, including the MAPK/NF-κB cascade [40]. Furthermore, rosmarinic acid has demonstrated antioxidant effects in vivo, which appear to be predominantly mediated via indirect mechanisms. These mechanisms include the upregulation of endogenous antioxidant enzymes such as catalase, glutathione peroxidase, and superoxide dismutase, as well as the enhancement of glutathione levels. Additionally, rosmarinic acid modulates key redox-sensitive signaling pathways, notably the Nrf2 pathway, along with insulin/insulin-like growth factor signaling (IIS) and MAPK cascades. Through these pathways, rosmarinic acid enhances the cellular antioxidant defense system, thereby mitigating oxidative stress and associated tissue damage [39,41,42].

Our phytochemical analysis revealed that AM and AS extracts are valuable sources of tilianin and phenolic acids (rosmarinic acid, chlorogenic acid)—bioactive compounds with well-documented pharmacological activities in inflammatory conditions and oxidative stress-associated disease models [14,39].

### 3.2. Quantification of Total Polyphenols, Flavonoids, and Caffeic Acid Derivatives in Agastache sp. Extracts

Our study revealed notable differences in the polyphenolic content between the two *Agastache* sp. extracts. The AM extract exhibited higher total phenolic content and total caffeic acid derivative levels compared to the AS extract, whereas the AS extract was characterized by a greater total flavonoid content (Table 3).

According to the existing literature, TFC values reported for AM were 54.47 ± 0.73% for subsp. *mexicana* and 25.29 ± 0.49% for subsp. *xolocotziana* (with quercetin used as the reference compound and results expressed as a percentage of the average), while TPC reached 320 ± 4.7 mg GAE/g dry plant extract for subsp. *mexicana*, and 162.3 ± 1.23 mg GAE/g dry plant extract for subsp. *xolocotziana*, indicating significant variability among subspecies [43]. In other *Agastache* sp. extracts, TPC values of 38.11 ± 0.88 mg GAE/g extract [32] and 38.9 ± 1.7 mg GAE/g extract [44] have been reported for *A. rugosa*. Additionally, Oh et al. reported a TFC of 22.8 mg naringin equivalents/g extract in *A. rugosa* [45].

The total polyphenolic content was determined by the Folin–Ciocalteu method, which measures the reducing capacity of the extract and may respond variably to different phenolic subclasses [25]. Therefore, the sum of flavonoids and caffeic acid derivatives quantified by specific assays may sometimes exceed the total polyphenol value. This discrepancy arises from methodological differences and does not reflect an actual contradiction in polyphenol composition.

Although direct comparisons between species are limited by differences in extraction procedures and measurement units, our results suggest that AM and AS extracts may serve as valuable sources of polyphenols—particularly flavonoids and caffeic acid derivatives—phytochemicals known for their well-documented antioxidant activity [46].

### 3.3. Assessment of In Vitro Antioxidant Activity

The in vitro antioxidant capacity of the two *Agastache* sp. extracts was evaluated using the DPPH and FRAP assays. The results of our study demonstrated that both extracts exhibited significant antioxidant potential against the tested free radicals (Table 4). AM extract showed slightly higher DPPH radical scavenging activity compared to the AS extract. Similarly, in the FRAP assay, AM extract exhibited higher ferric reducing antioxidant power than AS extract.

These findings are consistent with previous studies on other *Agastache* species. For instance, research on *A. rugosa* demonstrated that DPPH radical scavenging activity varied across different flowering stages, with the highest antioxidant activity observed during the fully open flowering stage [47]. Several studies have reported a positive correlation between the total phenolic content of *A. rugosa* extracts and their antioxidant activity, suggesting that the observed effects are primarily attributable to phenolic compounds [47,48,49]. Notably, tilianin and rosmarinic acid—major constituents of the extract—have been extensively documented to possess potent antioxidant properties across various experimental models [14,15,16,39].

For example, a study by Desta et al. identified 18 polyphenolic compounds in different parts of *A. rugosa* and demonstrated that antioxidant activity, assessed by assays such as DPPH and ABTS, increased proportionally with polyphenol content [48]. Similarly, research on honey derived from *A. rugosa* revealed a positive correlation between total polyphenol content and antioxidant activity, as measured by DPPH radical scavenging capacity [49].

In our study, the higher TPC observed for AM extract corresponds with its enhanced antioxidant activity compared to AS extract. These findings are in accordance with the idea that phenolic compounds are key contributors to the antioxidant potential of extracts [47,48,49]. However, it is important to consider that antioxidant activity can also be influenced by the specific nature of the phenolic constituents and the possible synergistic effects between them [50]. Therefore, further characterization of the phenolic profiles could provide a more comprehensive understanding of the antioxidant mechanisms underlying the activity of AM and AS extracts.

### 3.4. Investigation of In Vivo Antioxidant and Anti-Inflammatory Effects

The in vivo antioxidant and anti-inflammatory effects of AM and AS extracts were investigated using a rat model of acute inflammation induced by turpentine oil. To assess the biochemical response to treatment, multiple serum markers of oxidative stress were quantified (Table 5).

The induction of inflammation (INFL group) resulted in a significant increase in oxidative stress markers—OSI (*p* < 0.01), TOS, and NO (*p* < 0.05)—compared to the control group. Simultaneously, antioxidant defense markers such as TAC (*p* < 0.001) and SH (*p* < 0.05) were significantly decreased.

The AS extract demonstrated a pronounced antioxidant and anti-inflammatory effect, particularly at higher doses. The administration of AS1 significantly decreased TOS and OSI values (*p* < 0.001), while AS2 induced moderate reductions (*p* < 0.05), and AS3 showed no statistically significant effect. Interestingly, TAC levels were significantly elevated by both AS2 and AS3 (*p* < 0.001), with a milder increase observed at AS1 (*p* < 0.05). MDA concentrations were most substantially reduced at AS2 (*p* < 0.001), followed by significant decreases at AS1 and AS3 (*p* < 0.01). NO levels were significantly reduced only at the intermediate dose (*p* < 0.001), whereas all three doses resulted in significant increases in SH levels (*p* < 0.05), reflecting enhanced antioxidant capacity.

In contrast, the AM1 and AM2 extracts produced significant increases in TAC (*p* < 0.001), and a less pronounced effect at AM3 (*p* < 0.05). SH levels were significantly elevated only by AM1 (*p* < 0.05). However, no statistically significant changes in TOS or OSI were observed following AM treatment. MDA levels were significantly reduced across all doses, with the most marked decrease at AM1 (*p* < 0.001), and moderate reductions at AM2 and AM3 (*p* < 0.01).

To establish a comparative baseline for antioxidant and anti-inflammatory activity, diclofenac (a non-steroidal anti-inflammatory drug) and Trolox (a water-soluble vitamin E analog) were used as positive controls. Diclofenac administration significantly modulated TOS, OSI, TAC, and NO levels (*p* < 0.001), and moderately reduced MDA concentrations (*p* < 0.01), consistent with its mechanism of action via non-selective cyclooxygenase (COX) inhibition, which limits prostaglandin synthesis and mitigates inflammatory responses [51]. As inflammation and oxidative stress are closely interconnected processes [52,53], the observed modulation of oxidative markers in our study could be linked to its anti-inflammatory activity.

On the other hand, Trolox significantly increased TAC levels (*p* < 0.001), moderately elevated SH concentrations (*p* < 0.05), and effectively reduced MDA (*p* < 0.01) and TOS (*p* < 0.05) levels. As a potent antioxidant, Trolox acts primarily by directly scavenging reactive oxygen species (ROS) and stabilizing free radicals, thereby enhancing endogenous antioxidant defenses [54]. The inclusion of both diclofenac and Trolox as reference compounds provides a comparative context for investigating the dual antioxidant and anti-inflammatory potential of AM and AS extracts.

The observed biological effects are likely attributable to the major polyphenolic constituents identified in the extracts—tilianin and rosmarinic acid—which have been extensively documented for their pharmacological properties [14,15,16,38,39,40,42]. Tilianin has been shown to enhance the activity of key antioxidant enzymes such as superoxide dismutase (SOD), catalase (CAT), and glutathione peroxidase (GPx), while simultaneously reducing MDA levels in diabetic retinopathy models [55]. Moreover, it modulates oxidative stress and apoptosis via inhibition of the MAPK signaling pathway, which plays a central role in inflammatory regulation [56]. Rosmarinic acid exhibits anti-inflammatory efficacy through the modulation of NF-κB and MMP-9 activity, while also reducing hepatic and cellular injury markers such as GOT, GPT, and LDH in vivo [57].

Findings from previous studies on other *Agastache* sp. extracts or essential oils further support the results of our study. Moon et al. demonstrated that *A. rugosa* extract acts as an agonist of TRPA1 (transient receptor potential ankyrin 1) and TRPV1 (transient receptor potential vanilloid 1) receptors, which are implicated in inflammation-related signaling [58]. Additionally, the essential oil of *A. rugosa* was shown to significantly suppress the production of pro-inflammatory cytokines, including TNF-α and IL-6, in experimental arthritis models [59]. Based on the literature reviewed, there are a limited number of studies investigating the anti-inflammatory potential of AM and AS extracts, which highlights the originality and scientific relevance of our findings.

In summary, our study indicates that both AM and AS extracts exhibit significant anti-inflammatory activity, mediated through the modulation of oxidative stress parameters. These effects may be primarily attributed to their complex polyphenolic profiles, particularly the presence of tilianin and rosmarinic acid in high concentrations. However, further studies are required to elucidate the molecular mechanisms involved in the manifestation of the anti-inflammatory activity. Future research should focus on the modulation of specific pro-inflammatory mediators to better characterize the pathways through which these plant extracts exert their effects.

### 3.5. Investigation of In Vivo Cardioprotective Activity

The in vivo cardioprotective potential of AM and AS extracts was assessed using an isoprenaline-induced acute myocardial infarction model in rats. The therapeutic response was assessed through the evaluation of oxidative stress parameters (TOS, OSI, TAC, MDA, SH, and NOx), as well as biochemical markers indicative of myocardial injury (GOT, GPT, CK-MB) (Table 6).

Compared to the control group, the induction of MI resulted in increased serum levels of GOT, GPT (*p* < 0.01), and CK-MB (*p* < 0.001). Moreover, a marked oxidative imbalance was observed, characterized by increased levels of TOS, OSI (*p* < 0.001), and NOx (*p* < 0.05), along with a concomitant decrease in TAC and SH levels (*p* < 0.01).

Compared to the isoprenaline group, pretreatment with AS1 extract led to a statistically significant reduction in TOS and OSI levels (*p* < 0.001). A moderate yet significant decrease was observed following administration of AS2 (*p* < 0.05), while AS3 did not produce a statistically significant effect (*p* > 0.05). Pretreatment with AS1 led to a highly significant increase in TAC levels (*p* < 0.001), while AS2 and AS3 produced moderate but significant increases (*p* < 0.01). Regarding lipid peroxidation, MDA levels were most markedly reduced by AS1 and AS3 (*p* < 0.001), with AS2 exhibiting a less pronounced effect (*p* < 0.01). A significant reduction in NO concentrations was observed only following the administration of AS1 (*p* < 0.001). Moreover, all doses of AS significantly elevated SH levels, with the most notable effect recorded for AS2 (*p* < 0.01). In terms of cardiac injury markers, only AS1 significantly decreased GOT and GPT levels (*p* < 0.01), as well as CK-MB levels (*p* < 0.001), whereas the lower doses did not produce statistically significant changes in these parameters (*p* > 0.05).

Pretreatment with AM extract demonstrated dose-dependent modulation of oxidative stress and cardiac injury biomarkers. At the highest concentration (AM1), significant changes were observed in MDA and SH levels (*p* < 0.01), as well as in TOS, TAC, and OSI (*p* < 0.05), while NO values remained statistically unchanged (*p* > 0.05). Furthermore, AM1 significantly reduced serum GOT and GPT (*p* < 0.05), along with a marked decrease in CK-MB levels (*p* < 0.01). The intermediate dose (AM2) also produced significant effects, with reductions in MDA (*p* < 0.05), TOS, and OSI (*p* < 0.001), coupled with increased TAC and SH levels (*p* < 0.01). Correspondingly, GOT (*p* < 0.05), GPT (*p* < 0.01), and CK-MB (*p* < 0.001) levels were significantly decreased. The AM3 dose was still associated with measurable antioxidant and cardioprotective activity, as evidenced by reduced MDA, TOS, and OSI levels (*p* < 0.01), and a moderate increase in TAC (*p* < 0.01), although changes in SH were not statistically significant (*p* > 0.05). Interestingly, a significant reduction in NO levels was observed only at this dose (*p* < 0.01), suggesting a potential non-linear dose-response relationship in nitric oxide modulation.

Comparative analysis of the two extracts revealed that both AM and AS exhibited significant antioxidant and cardioprotective effects, with some differences in their activity profiles. At the highest tested dose, both extracts significantly reduced markers of oxidative stress such as MDA, TOS, and OSI, and increased antioxidant defenses (TAC, SH), although AS1 was slightly more effective in enhancing TAC (*p* < 0.001 vs. *p* < 0.05) and reducing NO levels, an effect not observed for AM1 (*p* > 0.05). Regarding cardiac injury markers, both AS1 and AM1 significantly decreased GOT, GPT, and CK-MB, with AS1 showing slightly stronger effects on CK-MB (*p* < 0.001 vs. *p* < 0.01 for AM1).

In this experimental model of isoprenaline-induced MI, Trolox was used as a positive control due to its well-established antioxidant capacity [54]. The administration of Trolox led to statistically significant improvements in oxidative stress biomarkers, with reductions in TOS and OSI (*p* < 0.05), MDA (*p* < 0.05), and NO (*p* < 0.01), alongside significant increases in TAC and SH levels (*p* < 0.01). Moreover, Trolox exerted cardioprotective effects, as evidenced by the reduction in serum GOT, GPT (*p* < 0.05), and CK-MB (*p* < 0.001) levels. These findings confirm the validity of the experimental model and establish a reference point for evaluating the efficacy of the AM and AS extracts, while also suggesting that the antioxidant and cardioprotective effects of AM and AS extracts may be partially mediated by mechanisms similar to those of standard antioxidants like Trolox.

Tilianin, the major phenolic constituent identified in both AM and AS extracts, has been extensively studied for its cardioprotective potential. Several in vitro and in vivo investigations have demonstrated that tilianin exerts protective effects against myocardial injury by modulating key pathways involved in oxidative stress and inflammation [14,15,38,55,56]. For instance, tilianin has been reported to enhance the activity of endogenous antioxidant enzymes (SOD, CAT, GP_X_), reduce lipid peroxidation (MDA levels), and suppress nitric oxide overproduction [16,60,61]. Moreover, tilianin has been shown to inhibit pro-apoptotic and pro-inflammatory signaling pathways, including NF-κB and MAPK, contributing to improved myocardial histology and biochemical profiles in models of ischemia-reperfusion injury [38,60]. These findings provide a mechanistic basis for the significant improvements observed in oxidative stress markers (TOS, TAC, OSI, MDA, NO, SH) and cardiac injury biomarkers (GOT, GPT, CK-MB) following the administration of AM and AS extracts in our study.

In addition to tilianin, rosmarinic acid was identified in significant quantities in both extracts. Rosmarinic acid is a well-characterized phenolic compound known for its potent antioxidant and anti-inflammatory properties [39,40,41,42,57]. Previous research has demonstrated its ability to attenuate myocardial injury through the reduction in oxidative stress, enhancement of nitric oxide bioavailability, and inhibition of key inflammatory mediators such as TNF-α and IL-6 [62,63].

A comparison between our findings and the existing literature highlights that, although the extract of *A. mexicana* has been investigated in several studies emphasizing its cardiovascular benefits—such as vasorelaxant and antihypertensive effects primarily attributed to bioactive compounds like tilianin [23,64,65,66]—there remains a significant gap in the literature regarding the cardioprotective properties of *A. scrophulariifolia* extract. To our knowledge, this study is the first to investigate the cardioprotective effects of *A. scrophulariifolia* extract in an in vivo model of isoprenaline-induced myocardial injury. Consequently, our results provide novel insights into the therapeutic potential of this species and simultaneously broaden the existing knowledge on *A. mexicana*.

Although a degree of mortality was recorded in specific experimental groups (Group ISO: 1, Trolox: 2, AS2: 3, AS3: 3, AM1: 3, AM2: 1, AM3: 3), it occurred during or shortly after myocardial infarction induction, a procedure known for its severity and inherent risk of fatal outcomes in animal models. Importantly, no signs of acute toxicity were noted prior to MI induction. Moreover, the biochemical parameters assessed—namely GOT, GPT, and CK-MB—showed favorable modulation under extract treatment, supporting the absence of hepatotoxic or cardiotoxic effects. Notably, mortality was also recorded in control groups after myocardial infarction induction, further indicating that mortality was related to the procedure itself rather than to the administration of the extracts. While a formal acute toxicity study was not included in this work, these findings suggest a protective rather than a toxic influence of the extracts.

Therefore, the results of our study highlight the cardioprotective potential of lyophilized extracts from *A. mexicana* and *A. scrophulariifolia*, demonstrated by their capacity to attenuate oxidative stress and reduce biochemical markers associated with myocardial injury. Both extracts significantly improved antioxidant status—reflected by elevated TAC and SH levels—and decreased oxidative markers such as TOS, OSI, MDA, and NO, alongside marked reductions in serum GOT, GPT, and CK-MB levels. These beneficial effects are likely mediated by the extracts’ polyphenolic composition, particularly the presence of tilianin and rosmarinic acid, compounds previously reported to exhibit antioxidant and cardioprotective activities [14,39,57]. Collectively, these findings support the potential application of these plant extracts as promising natural sources for the management of oxidative stress-related cardiac injuries. A potential limitation of the study is the inherent biological variability among individual animals, which may influence the observed responses and contribute to data dispersion despite standardized experimental conditions.

## 4. Limitations

A notable mortality rate was recorded in the infarct group, most likely due to the severity of the isoprenaline-induced myocardial injury. This reduced the final sample size and may affect the statistical robustness of the results; however, the remaining data still support the overall conclusions of the study.

## 5. Conclusions

This study offers a comprehensive analysis of the polyphenolic composition of *A. mexicana* and *A. scrophulariifolia* lyophilized extracts. Both qualitative and quantitative assessments revealed significant levels of total polyphenols, flavonoids, and caffeic acid derivatives, with tilianin and rosmarinic acid identified as major constituents. In vitro antioxidant assays (DPPH and FRAP) confirmed a notable free-radical-scavenging capacity for both extracts.

In vivo, we investigated the antioxidant, anti-inflammatory, and cardioprotective properties of the extracts in rat models of turpentine-induced inflammation and isoprenaline-induced myocardial infarction. Treatment with both extracts led to significant modulation of oxidative stress markers, characterized by a reduction in pro-oxidant parameters (TOS, OSI, MDA, NO) and an enhancement of antioxidant markers (TAC, SH). Additionally, the extracts markedly decreased serum levels of GOT, GPT, and CK-MB, suggesting a protective effect against myocardial injury.

The results of our study show that *A. mexicana* and *A. scrophulariifolia* extracts represent valuable sources of bioactive phytoconstituents—particularly tilianin and rosmarinic acid—with significant pharmacological relevance. The in vivo validation of their effects further reinforces the potential of these extracts in the management of oxidative stress-related conditions. Further investigations are necessary to isolate and characterize the individual active compounds and describe their mechanisms of action. Nonetheless, the present study highlights the therapeutic potential of both extracts and provides novel insights, especially regarding *A. scrophulariifolia*, for which cardioprotective properties have not been previously documented.

## Figures and Tables

**Table 1:** The LC-MS gradient.

Time (min)	Methanol (%)	Water (%)	2%Formic Acid in Water (%)
0.00	5	90	5
3.00	15	70	15
6.00	15	70	15
9.00	21	58	21
13.00	21	58	21
18.00	30	41	29
22.00	30	41	29
26.00	50	0	50
29.00	50	0	50
29.01	5	90	5
35.00	5	90	5

**Table 2:** Polyphenolic compounds identified in *A. mexicana* and *A. scrophulariifolia* extracts.

Compound	Retention Time (min)	*m*/*z* and Main Transitions	Detection Limit (μg/mL)	Quantification Limit (μg/mL)	*A. mexicana* Extract (μg/mL)	*A. scrophulariifolia* Extract (μg/mL)
Gallic Acid	7.0	168.9 > 125.0	1.90	2.90	3.6 ± 0.05	4.0 ± 0.07
Chlorogenic Acid	11.9	353.0 > 191.0	5.00	8.00	4005.2 ± 35.42	3362.3 ± 5.47
Vitexin	13.0	179.1 > 123.0	1.30	2.00	2.5 ± 0.03	2.5 ± 0.05
Luteolin-7-O-glucoside	13.6	317.0 > 179.0	3.00	4.00	677.9 ± 8.49	762.1 ± 10.78
Caffeic Acid	13.8	179.0 > 135.0	3.20	4.80	189.7 ± 1.94	194.3 ± 3.21
Trans-*p*-coumaric Acid	17.5	163.0 > 119.0	2.50	4.90	28.7 ± 0.31	23.7 ± 0.51
Quercetin	18.4	431.0 > 311.0	0.80	1.10	2.5 ± 0.03	1.1 ± 0.02
Kaempferol	19.9	447.0 > 284.9	0.80	1.20	470.3 ± 7.14	397.9 ± 7.41
Naringenin	20.2	609.0 > 300.0	0.60	0.90	2.5 ± 0.03	2.3 ± 0.02
Tilianin	20.2	447.1 > 285.0	9.10	13.60	51,635.8 ± 278.21	55,574.8 ± 411.21
Esculetin	20.3	463.1 > 300.0	2.90	5.80	990.8 ± 12.17	748.7 ± 10.78
Rosmarinic Acid	21.4	358.9 > 161.0	0.10	0.20	5910.3 ± 99.41	4263.3 ± 59.24
Salicylic Acid	23.5	137.0 > 93.0	1.50	2.00	34.7 ± 0.72	47.9 ± 1.07
Myricetin	25.4	300.9 > 151.0	0.60	0.90	132.5 ± 1.87	145.1 ± 2.78
Luteolin	26.2	271.0 > 119.0	0.05	0.07	21.6 ± 0.27	13.0 ± 0.27
Hyperoside	26.8	287.0 > 153.0	0.60	0.90	18.9 ± 0.24	21.0 ± 0.37
Chrysin	27.0	301.0 > 164.0	3.00	5.00	4.5 ± 0.04	4.5 ± 0.07
Ellagic Acid	27.2	301.0 > 185.0	3.00	5.00	72.7 ± 1.21	449.5 ± 7.49
Hesperetin	27.9	285.0 > 187.0	3.00	5.00	9.9 ± 0.11	11.5 ± 0.17
Apigenin	28.1	269.0 > 117.0	0.20	0.30	43.5 ± 0.74	26.0 ± 0.87
Carnosol	29.7	253.0 > 143.0	1.00	2.00	10.8 ± 0.11	6.5 ± 0.10
Acacetin	30.0	283.1 > 268.0	0.20	0.30	6.6 ± 0.09	6.1 ± 0.07
Rutoside	30.7	329.1 > 285.1	4.00	6.00	7.8 ± 0.10	151.7 ± 2.45
Carnosic Acid	32.0	331.2 > 285.1	4.00	6.00	51.4 ± 0.84	54.3 ± 1.07

Note: d.w.—dry weight. Each value is the mean of three replicates ± SD.

**Table 3:** Quantitative analysis of TPC, TFC, and TCADC in *A. mexicana* and *A. scrophulariifolia* extracts.

Extract	TPC (mg GAE/g d.w.)	TFC (mg RE/g d.w.)	TCADC (mg CAE/g d.w.)
*A. mexicana*	51.33 ± 1.53	9.73 ± 0.15	36.17 ± 0.76
*A. scrophulariifolia*	36.33 ± 1.53	11.73 ± 1.07	27.38 ± 0.85

Note: TPC: total polyphenols content; TFC: total flavonoids content; TCADC: total caffeic acid derivatives content; GAE: gallic acid equivalents; d.w.: dry weight; RE: rutoside equivalents; CAE: caffeic acid equivalents. Each value is the mean of three replicates ± SD.

**Table 4:** Assessment of in vitro antioxidant activity of *A. mexicana* and *A. scrophulariifolia* extracts.

Extract	DPPH Assay IC_50_ (µg/mL)	FRAP Assay (µM of TEs/100 mL of Extract)
*A. mexicana*	65.99 ± 1.21	2566.71 ± 267.55
*A. scrophulariifolia*	68.64 ± 2.48	1688.76 ± 212.32

Note: IC_50_: half-maximal inhibitory concentration; TEs: Trolox equivalents. Each value is the mean of three replicates ± SD.

**Table 5:** Modulatory Effects of *A. scrophulariifolia* and *A. mexicana* Extracts on Serum Oxidative Stress Markers in a Rat Model of Turpentine Oil-Induced Inflammation.

Group	TOS (µM H_2_O_2_ E/L)	OSI	TAC (mM TE/L)	NOx (µM/L)	MDA (μM/L)	SH (µM/L)
CONTROL	10.81 *±* 0.51	10.33 ± 0.49	1.046 ± 0.003	38.05 ± 4.59	6.68 ± 10.21	628.2 ± 55.41
INFL	13.06 *±* 0.59 *	12.62 ± 0.57 **	1.035 ± 0.001 ***	43.31 ± 1.39 *	5.19 ± 0.33	520.6 ± 66.96 *
TROLOX	12.08 ± 0.35 **^#^	11.55 ± 0.33 **	1.046 ± 0.002 ^###^	38.91 ± 8.30	4.21 ± 0.29 ^##^	675.8 ± 97.02 ^#^
DICLO	11.33 *±* 0.38 ^###^	10.82 ± 0.32 ^###^	1.048 ± 0.004 ^###^	37.79 ± 1.65 ^###^	4.34 ± 0.21 ^##^	490.2 ± 44.37 *
AS1	11.35 *±* 0.18 ^###^	10.84 ± 0.16 ^###^	1.047 ± 0.003 ^#^	45.49 ± 2.02 *	4.06 ± 0.44 ^##^	625.4 ± 33.17 ^#^
AS2	12.14 *±* 0.49 *^#^	11.54 ± 0.52 *^#^	1.052 ± 0.006 ^###^	31.26 ± 1.96 *^###^	3.84 ± 0.23 ^###^	608.6 ± 48.11 ^#^
AS3	12.64 *±* 0.30 **	12.10 ± 0.49 **	1.045 ± 0.002 ^###^	48.37 ± 2.85 **^##^	4.30 ± 0.27 ^##^	611.4 ± 48.85 ^#^
AM1	13.08 *±* 0.61 **	12.43 ± 0.53 **	1.052 ± 0.005 ^###^	47.77 ± 3.77 *^#^	3.73 ± 0.17 ^###^	688.6 ± 101.72 ^#^
AM2	13.35 ± 0.54 **	12.71 ± 0.45 **	1.051 ± 0.005 ^###^	49.34 ± 5.40 *^#^	4.32 ± 0.29 ^##^	531.4 ± 87.38
AM3	12.74 ± 0.97 **	12.20 ± 0.89 **	1.044 ± 0.004 ^##^	39.11 ± 6.62	4.13 ± 0.35 ^##^	525.4 ± 60.07 *

Note: * *p* < 0.05, ** *p* < 0.01, *** *p* < 0.001 versus CONTROL; ^#^ *p* < 0.05, ^##^ *p* < 0.01, ^###^ *p* < 0.001 versus INFL. TOS—total oxidative status, OSI—oxidative stress index, TAC—total antioxidant capacity, NOx—total nitrites and nitrates, MDA—malondialdehyde, SH—total thiols, TEs—Trolox equivalents. Each experimental group included 5 animals. Treatment groups and administered doses: CONTROL: distilled water, 1 mL/rat/day, no inflammation induction; INFL: distilled water, 1 mL/rat/day + turpentine oil (6 mL/kg, i.m.); DICLO: diclofenac, 10 mg/kg/day + turpentine oil; TROLOX: Trolox, 50 mg/kg/day + turpentine oil; AS1: AS extract, 19 mg/mL, 1 mL/rat/day + turpentine oil; AS2: AS extract, 9.5 mg/mL, 1 mL/rat/day + turpentine oil; AS3: AS extract, 4.75 mg/mL, 1 mL/rat/day + turpentine oil; AM1: AM extract, 21 mg/mL, 1 mL/rat/day + turpentine oil; AM2: AM extract, 10.5 mg/mL, 1 mL/rat/day + turpentine oil; AM3: AM extract, 5.25 mg/mL, 1 mL/rat/day + turpentine oil. Values are expressed as mean ± SD (n = 5).

**Table 6:** Investigation of the In Vivo Cardioprotective Effects of *A. mexicana* and *A. scrophulariifolia* Lyophilized Extracts in an Isoprenaline-Induced Myocardial Infarction Rat Model.

Group	TOS (µM H_2_O_2_ E/L)	OSI	TAC (mM TE/L)	NO_X_ (μM/L)	MDA (μM/L)	SH (μM/L)	GOT (U/L)	GPT (U/L)	CK-MB (U/L)
CONTROL	10.8 ± 0.51	10.33 ± 0.49	1.046 ± 0.003	38.05 ± 4.59	6.68 ± 10.21	628.2 ± 55.41	40.20 ± 6.66	47.29 ± 4.34	7.71 ± 0.68
ISO	12.96 ± 0.32 ***	12.50 ± 0.33 ***	1.036 ± 0.003 **	44.37 ± 2.77 *	3.39 ± 0.15	480.5 ± 30.74 **	62.26 ± 7.11 **	62.74 ± 5.03**	11.20 ± 0.67 ***
TROLOX	12.20 ± 0.31 **^#^	11.63 ± 0.32 **^#^	1.049 ± 0.003 ^##^	36.79 ± 1.30 ^##^	2.82 ± 0.42 ^#^	673.67 ± 63.13 ^##^	47.63 ± 6.00 ^#^	50.30 ± 5.81 ^#^	7.82 ± 0.52 ^###^
AM1	11.73 ± 0.34 ^#^	11.23 ± 0.31 ^#^	1.044 ± 0.001 ^#^	41.39 ± 0.72	2.54 ± 0.09 ^##^	629 ± 33.94 ^##^	40.41 ± 2.99 ^#^	44.28 ± 4.06 ^#^	8.34 ± 0.00 ^##^
AM2	11.63 ± 0.29 *^###^	11.10 ± 0.25 *^###^	1.049 ± 0.005 ^##^	40.47 ± 4.72	2.65 ± 0.37 ^#^	552 ± 20.30 *^##^	46.88 ± 7.04 ^#^	47.39 ± 5.89 ^##^	8.34 ± 0.43 ^###^
AM3	11.54 ± 0.34 ^##^	10.98 ± 0.30 ^##^	1.051 ± 0.002 ^##^	28.16 ± 1.70 ^##^	2.70 ± 0.15 ^##^	476 ± 43.84 *	45.95 ± 1.87 ^#^	49.14 ± 0.35 ^#^	8.86 ± 0.00 ^#^
AS1	11.01 ± 0.26 ^###^	10.55 ± 0.25 ^###^	1.044 ± 0.001 ^###^	30.41 ± 3.24 *^###^	2.69 ± 0.12 ^###^	610.2 ± 42.18 ^#^	42.89 ± 6.71 ^##^	45.35 ± 4.98 ^##^	7.92 ± 0.44 ^###^
AS2	11.97 ± 0.14 *^#^	11.41 ± 0.15 *^#^	1.049 ± 0.002 ^##^	48.16 ± 2.24	2.6 ± 0.18 ^##^	711 ± 59.40 ^##^	70.25 ± 7.84 **	71.59 ± 2.82 ***	9.90 ± 0.00
AS3	12.79 ± 0.20 **	12.20 ± 0.16 **	1.049 ± 0.003 ^##^	40.57 ± 5.82	2.35 ± 0.06 ^###^	619 ± 28.28 ^##^	64.71 ± 2.99 **	65.23 ± 5.82 **	9.90 ± 0.00

Note: * *p* < 0.05, ** *p* < 0.01, *** *p* < 0.001 versus CONTROL; ^#^ *p* < 0.05, ^##^ *p* < 0.01, ^###^ *p* < 0.001 versus ISO. TOS—total oxidative status, OSI—oxidative stress index, TAC—total antioxidant capacity, NOx—total nitrites and nitrates, MDA—malondialdehyde, SH—total thiols, TEs—Trolox equivalents, U/L—units/liter, GOT—Glutamate Oxaloacetate Transaminase, GPT—Glutamate Pyruvate Transaminase, CK-MB—Creatine Kinase-MB Isoenzyme. Each experimental group included 5 animals. Treatment groups and administered doses: CONTROL: distilled water, 1 mL/rat/day, no myocardial infarction induction; ISO: distilled water, 1 mL/rat/day + isoprenaline (150 mg/kg, s.c.) on days 8 and 9; TROLOX: Trolox, 50 mg/kg/day, 1 mL/rat/day + isoprenaline (150 mg/kg, s.c.) on days 8 and 9; AS1: AS extract, 19 mg/mL, 1 mL/rat/day + isoprenaline; AS2: AS extract, 9.5 mg/mL, 1 mL/rat/day + isoprenaline; AS3: AS extract, 4.75 mg/mL, 1 mL/rat/day + isoprenaline; AM1: AM extract, 21 mg/mL, 1 mL/rat/day + isoprenaline; AM2: AM extract, 10.5 mg/mL, 1 mL/rat/day + isoprenaline; AM3: AM extract, 5.25 mg/mL, 1 mL/rat/day + isoprenaline. Values are expressed as mean ± SD (n = 5). Values with an SD of 0.00 correspond to replicate measurements with minimal variability.

## Data Availability

The raw data supporting the conclusions of this study are available upon request.

## Abbreviations

The following abbreviations are used in this manuscript:

HPLC-MS	High-Performance Liquid Chromatography–Mass Spectrometry
DPPH	2,2-Diphenyl-1-picrylhydrazyl
FRAP	Ferric Reducing Antioxidant Power
IC_50_	Half Maximal Inhibitory Concentration
TOS	Total Oxidant Status
OSI	Oxidative Stress Index
TAC	Total Antioxidant Capacity
MDA	Malondialdehyde
NO	Nitric Oxide
SH	Sulfhydryl Groups
GOT	Glutamate Oxaloacetate Transaminase
GPT	Glutamate Pyruvate Transaminase
CK-MB	Creatine Kinase-MB Isoenzyme
CVD	Cardiovascular Disease
LDL	Low-Density Lipoprotein
CRP	C-Reactive Protein
ROS	Reactive Oxygen Species
LDH	Lactate Dehydrogenase
ATP	Adenosine Triphosphate
NAD	Nicotinamide Adenine Dinucleotide
SOD	Superoxide Dismutase
AM	*Agastache mexicana*
AS	*Agastache scrophulariifolia*
TPC	Total Phenolic Content
GAE	Gallic Acid Equivalents
TFC	Total Flavonoids Content
RE	Rutoside Equivalents
TCADC	Total Caffeic Acid Derivatives Content
CAE	Caffeic Acid Equivalents
TPTZ	2,4,6-Tripyridyl-s-triazine
ISO	Isoprenaline
MAPK	Mitogen-Activated Protein Kinase
NF-KB	Nuclear Factor Kappa B
TE	Trolox Equivalents
INFL	Inflammation
CAT	Catalase
GPx	Glutathione Peroxidase
MMP-9	Matrix Metalloproteinase-9
TRPA1	Transient Receptor Potential Ankyrin 1
TRPV1	Transient Receptor Potential Vanilloid 1
TNF-alfa	Tumor Necrosis Factor-alpha
IL-6	Interleukin-6
MI	Myocardial Infarction
